# Effects of Dihydroartemisinin-Piperaquine Phosphate and Artemether-Lumefantrine on QTc Interval Prolongation

**DOI:** 10.1038/s41598-018-37112-6

**Published:** 2019-01-28

**Authors:** Christian Funck-Brentano, Antonella Bacchieri, Giovanni Valentini, Silvia Pace, Silva Tommasini, Pascal Voiriot, David Ubben, Stephan Duparc, Eric Evene, Mathieu Felices, Marco Corsi

**Affiliations:** 1 INSERM, CIC-1421 and UMR ICAN 1166, Sorbonne Université, Faculty of Medicine, AP-HP, Pitié-Salpêtrière Hospital, Department of Pharmacology and Clinical Investigation Center, Institute of Cardiometabolism and Nutrition (ICAN), F-75013 Paris, France; 2 Sigma-tau Industrie Farmaceutiche Riunite S.p.A., Pomezia, (Rome) Italy; 3 Cardiabase, Nancy, France; 4 Medicines for Malaria Venture, Geneva, Switzerland; 5 SGS-Aster, Paris, France; 6 PhinC development, Evry, France

## Abstract

QT/QTc interval prolongation reflects delayed cardiac repolarization which can lead to *Torsade de Pointes* and sudden death. Many antimalarial drugs prolong QT/QTc interval. However, due to confounding factors in patients with malaria, the precise extent of this effect has been found to be highly variable among studies. We compared the effects of dihydroartemisinin-piperaquine phosphate (DHA-PQP) and artemether-lumefantrine (A-L) on QT interval duration in healthy volunteers. In this randomized, parallel groups, active moxifloxacin- and placebo-controlled study, prolongation of the QT/QTc interval following treatment with DHA-PQP in fasted and fed condition and A-L in fed state was investigated in healthy subjects (n = 287; Clinicaltrials.gov: NCT01103830). DHA-PQP resulted in significant mean (95% confidence interval (CI)) maximum increases in QTc Fridericia (QTcF) of 21.0 ms (15.7, 26.4) for DHA-PQP fasted, 35.9 ms (31.1, 40.6) for DHA-PQP high-fat/low-caloric and 46.0 ms (39.6, 52.3) for DHA-PQP high-fat/high-caloric breakfast. For A-L, the largest difference from baseline relative to placebo was 9.9 ms (95% CI: 6.8, 12.9). Increases in QTcF related to maximum plasma concentrations of piperaquine. Moxifloxacin demonstrated assay sensitivity. Increases in QTcF following DHA-PQP and A-L were clinically relevant. Food increased piperaquine exposure and QTcF interval prolongation emphasizing the need to administer DHA-PQP in the fasting state.

## Introduction

Malaria is a disease that although preventable and tractable still caused approximately 425,000 mortalities in 2016, with most deaths occurring in Africa^1^. The World Health Organization recommends artemisinin-based combination therapies for the treatment of *Plasmodium falciparum* malaria^2^. Many antimalarial drugs including artemisinin-based combination therapies have been associated with prolongation of the corrected QT interval (QTc), which reflects a delay in ventricular repolarization during the cardiac cycle^3^. Delayed cardiac repolarization can lead to the development of ventricular tachyarrhythmias, most notably *Torsade de Pointes*, which can be self-terminating but can also degenerate into ventricular fibrillation leading to sudden death.

QT interval is highly influenced by heart rate, physiologically. Assessment of possible QT-prolonging effects of antimalarial drugs in patients is often hampered by the symptoms of malaria most notably fever^3^. In the acute phase of the disease, and in addition to fever, stress, anxiety and discomfort may lead to an increase in heart rate. In contrast, during the recovery phase and after starting treatment, heart rate decreases and QT interval lengthens. Therefore since the clinical condition of patients with malaria may induce changes in the QT interval, the effects of antimalarial treatments on cardiac repolarization are ideally studied in healthy subjects.

The fixed-dose artemisinin-based combination therapy of dihydroartemisinin (DHA) and piperaquine phosphate (PQP) is approved in the European Union for the treatment of uncomplicated *P. falciparum* malaria. Preclinical experiments showed that despite significant blockade of the human ether-a-go-go related channel (hERG), which plays a critical role in cardiac repolarization, DHA-PQP did not appear to induce effects characteristic of *Torsade de Pointes*, affect hERG trafficking or block sodium channels although it blocked slow-potassium ion currents^4^. However, in patients with malaria, treatment with DHA-PQP resulted in prolongation of the Fridericia corrected QT interval (QTcF) ranging from 7 to 45 ms^5–8^. Marked QTcF prolongations were also observed for artemisinin-based combination therapies (artemether-lumefantrine [A-L]: 22 ms^9^; artesunate + mefloquine: 18 ms^10^; artesunate + amodiaquine: 33 ms^9^.). In these studies, electrocardiograms (ECGs) were recorded at various single-time points when plasma drug concentrations were at trough or around presumed peak levels; therefore, the treatment effect on QT interval may have been under- or overestimated.

The present study was conducted in healthy subjects to carefully investigate prolongation of the QT/QTc interval following treatment with DHA-PQP and A-L at the maximum plasma concentrations of their respective active compounds and over the 24 hrs following the last day of drug administrations. The methodology employed was based on that used in thorough QTc studies, which are designed to detect small changes in QTcF^11^.

## Results

### Subject Disposition and Demographics

**Figure 1:**
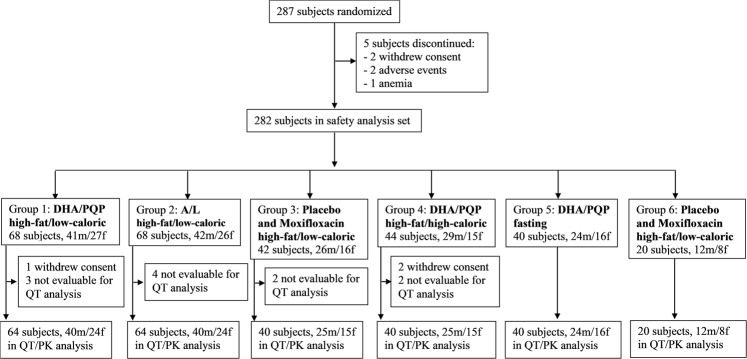
Flow chart of subject disposition. Group 1, dihydroartemisinin-piperaquine phosphate (DHA-PQP) low-caloric breakfast; Group 2, artemether-lumefantrine (A-L); Group 3, moxifloxacin; Group 4, DHA-PQP high-caloric breakfast; Group 5, DHA-PQP fasted; Group 6, moxifloxacin. m = male; f = female.

A total of 287 healthy Caucasian subjects were randomized and their disposition in the study is presented in Fig. 1. Five subjects were withdrawn from the study between Day –1 and Day 1. Two hundred and eighty-two subjects (174 men and 108 women) received at least one dose of study medication and 279 completed the study. Subjects providing a non-evaluable QT interval were replaced to maintain the pre-established sample size in each group. The mean [range] age and body mass index of the study population were 29.6 [18–50] years and 22.6 [18.0–28.2] kg/m^2^, respectively. Demographic variables (including age, gender, weight and height; data not shown) were similar across groups.

### ECG Analysis

Fridericia’s method was the most appropriate to correct the QT interval for changes in heart rate (data not shown) and was used throughout this study. In Group 3 treatment with moxifloxacin caused a prolongation in QTcF interval (Table 1); the maximum time-matched difference from placebo was 15.5 ms (90% confidence interval [CI]: 12.5, 18.4) at 4 h post-dose. In Group 6 moxifloxacin caused a QTcF prolongation of similar magnitude: 17.4 ms (90% CI: 13.3, 21.5) at 6 h post-dose.

**Table 1:** Treatment comparisons for maximum time-matched changes in QTcF.

Assay sensitivity analysis	Mean (SE)	90% CI	p-value
Moxifloxacin (Group 3) – placebo: largest mean effect at 4 h	15.5 (1.8)	12.5; 18.4	<0.0001
Moxifloxacin (Group 6) – placebo: largest mean effect at 6 h	17.4 (2.5)	13.3; 21.5	<0.0001
**Analysis of variance for treatment comparison**			
**Treatment Comparisons vs placebo**	**Mean (SE)**	**95% CI**	**p-value**
DHA-PQP fasted – placebo (Group 6)	21.0 (2.7)	15.7; 26.4	<0.0001
A-L – placebo (Group 3)	9.9 (1.5)	6.8; 12.9	<0.0001
DHA-PQP high-fat/low-caloric – placebo (Group 3)	35.9 (2.4)	31.1; 40.6	<0.0001
DHA-PQP high-fat/high-caloric – placebo (Group 3)	46.0 (3.2)	39.6; 52.3	<0.0001
**Other Treatment Comparisons**			
DHA-PQP high-fat/low-caloric – A-L	26.0 (2.0)	22.0; 30.0	<0.0001
DHA-PQP fasted – A-L	13.4 (1.9)	9.7; 17.1	<0.0001
DHA-PQP high-fat/low-caloric – DHA-PQP fasted	12.6 (2.6)	7.4; 17.8	<0.0001

SE = standard error; CI = confidence interval; DHA = dihydroartemisinin; PQP = piperaquine phosphate; A = artemether; L = lumefantrine.

**Figure 2:**
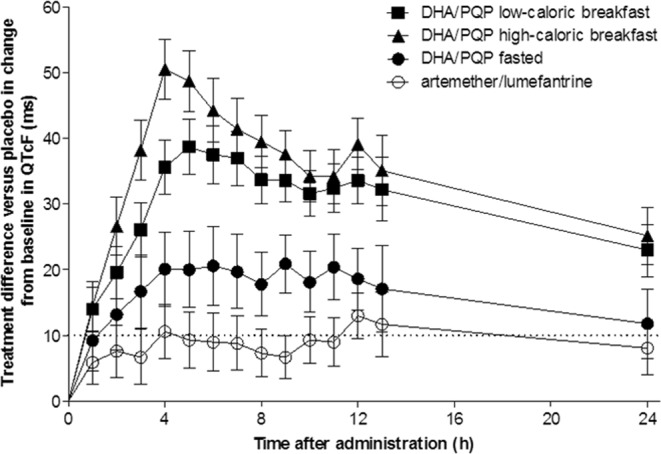
Difference from placebo (Day –1 of respective treatment) in mean change from baseline in QTcF on Day 3. Data are presented as mean and 90% confidence interval. The dotted horizontal line corresponds to the 10 ms threshold. DHA-PQP = dihydroartemisinin-piperaquine phosphate. Square = DHA-PQP low caloric breakfast; triangle = DHA-PQP high caloric breakfast; filled circle = DHA-PQP fasted; open circle = artemether-lumefantrine.

QTcF increased with DHA-PQP. Mean QTcF prolongation with DHA-PQP was >10 ms for at least 24 h post-dose regardless of food intake (Fig. 2). QTcF prolongation with DHA-PQP significantly increased after a high-fat/low-caloric breakfast compared with the fasted state. For A-L, the largest difference from baseline relative to placebo (Group 3, Day 3) was 9.9 ms (95% CI: 6.8, 12.9; p < 0.0001) and this was significantly smaller than the QT prolonging effect of DHA-PQP high-fat/low-caloric (i.e. the primary hypothesis was rejected). Other comparisons are shown in Table 1. Prolongation of QTcF was 33.8 ms for DHA-PQP and 20.4 ms for A-L, before allowing for placebo adjustment. Body weight did not significantly influence QTcF. In contrast, gender had a statistically significant effect on QTcF in all treatment groups except DHA-PQP fasted, with longer QTcF interval prolongations noted in women. QTcF change from baseline was linearly related to the Cmax and AUC of PQ (supplementary file).

Categorical analysis showed that two subjects (3.1%) in the A-L group and four subjects (10%) in the DHA-PQP fasted group had abnormal maximum absolute QTcF values in the range 450–480 ms. There were no subjects from either group with maximum absolute QTcF values ≥480 ms. No subjects in the DHA-PQP fasted and A-L groups experienced maximum time-matched changes from baseline for QTcF > 60 ms.

### Pharmacokinetics

Maximum plasma concentration (C_max_) of DHA was achieved rapidly with median time to C_max_ (t_max_) values within 1–2 h (Table 2). Piperaquine was absorbed more slowly with median t_max_ values 3–4 h. When DHA-PQP was administered concomitantly with a high-fat/low-caloric breakfast, plasma PQ C_max_ increased approximately 2-fold compared with the fasted state. Concomitant administration of DHA-PQP with a high-fat/high-caloric breakfast resulted in a 3-fold increase in C_max_ with PQ reaching supra-therapeutic exposure. Lumefantrine was absorbed slowly (median t_max_: approximately 6 h), with subsequent slow formation of its desbuthyl metabolite (median t_max_: 7 h). Artemether plasma concentrations were below the limit of detection of the assay at all time-points in nine subjects. The median t_max_ value for its metabolite, DHA, was similar (median t_max_: 2 h) to that described after DHA-PQP administration, although C_max_ was lower.

**Table 2:** Pharmacokinetic variables of dihydroartemisinin (DHA), piperaquine (PQ), artemether, lumefantrine, desbuthyl-lumefantrine and moxifloxacin on treatment Day 3 or Day 4

Treatment	Analyte	C_max_	N	t_max_	N	AUC^a^	N	t_1/2_	N
		(ng/mL)		(h)		(ng·h/mL)		(h)	
DHA-PQP high-fat/low-caloric breakfast (Group 1)	DHA PQ	242 (39) 923 (41)	64 64	2 [1–4] 4 [2–12]	64 64	655 (34) 8639 (38)	64 64	1.38 (42) NE	64
DHA-PQP high-fat/high-caloric breakfast (Group 4)	DHA PQ	224 (40) 1390 (31)	40 40	2 [1–4] 4 [2–7]	40 40	656 (33) 12449 (33)	40 40	1.44 (38) NE	40
DHA-PQP fasted (Group 5)	DHA PQ	173 (41) 461 (58)	40 40	1 [1–4] 3 [2–6]	40 40	502 (35) 4868 (38)	39 40	1.52 (36) NE	39
Artemether-lumefantrine (group 2)	Artemether DHA Lumefantrine	34.1 (66) 85.8 (45) 19900 (38)	55 64 64	2 [1–5] 2 [1–5] 6 [4–9]	55 64 64	145 (45) 297 (34) 504173 (42)	24 45 64	NE 1.43 (49) NE	46
	Desbuthyl- lumefantrine	104 (52)	64	7 [0–13]	64	7639 (52)	64	NE	
Moxifloxacin (Group 3)	Moxifloxacin	2490 (18)	40	2 [1–4]	40	33379 (13)	12	8.15 (11)	12
Moxifloxacin (Group 6)	Moxifloxacin	2880 (15)	20	2 [1–4]	20	34984 (21)	8	8.03 (10)	8

Data are presented as mean (CV%) or, for t_max,_ as median [range].

^a^AUC_0–∞_ for DHA and moxifloxacin; AUC_0–24_ for PQ; AUC_0–t_ for artemether, lumefantrine and desbuthyl-lumefantrine.

C_max_ = maximum plasma concentration; t_max_ = time to C_max_; AUC_0–t_ = area under the time-concentration curve from time 0 to time of last quantifiable concentration; AUC_0–24 = _AUC from time 0 to 24 h post-dose; AUC_0–∞_ = AUC from time 0 to infinity; t½ = half-life; NE = not estimated due to limited sampling to preserve blinding.

## Discussion

In this study, clinically significant increases in QTcF were associated with DHA-PQP and A-L. In ICH E14 guideline an increase in QTcF is considered clinically relevant when the upper limit of the 90% CI of the maximum time-matched treatment difference from placebo in QTcF change from baseline exceeds 10 ms^11^. For all 3 DHA-PQP groups, even the lower limit of the 90% CI was well above this 10-ms limit whereas the effect of A-L was modest with only the upper limit of the 90% CI (12.0 ms) above 10 ms. Moxifloxacin was included in the present study as a positive control and administration of a single 400 mg dose resulted in an increase in QTcF of 15.5 and 17.4 ms. The increases in QTcF in the current study are in the high range among those reported for moxifloxacin in thorough QTc studies (10.2–21.0 ms)^12^, indicating that the subjects participating in the current study were sensitive to drug-induced QT prolongation.

Increases in QTcF observed following administration of DHA-PQP and A-L were clinically relevant. Treatment differences compared with placebo for DHA-PQP under fasted conditions showed increases similar to those of moxifloxacin. The QTcF prolongation observed for the DHA-PQP fasted group when the effect of placebo was not subtracted was higher than that determined in previous studies (33.8 ms for DHA-PQP in the current study vs. 19 to 23 ms for DHA-PQP in previous studies)^6,8,13^. This variability among studies emphasizes the need for a placebo control to accurately assess drug-induced QTc interval prolongation although it should be recognized that it is not feasible in patients with acute malaria.

Although several articles report on the effects of DHA-PQP on ventricular repolarization^5,6,14–16^, studies using high-standard of ECG recording and measurements over time for the specific evaluation of QTc prolongation after DHA-PQP are lacking. One study evaluated QTc prolongation after a full course of treatment with DHA-PQP in 56 patients with malaria^5^. No significant changes in mean QTcF occurred 4 h after the first administration of DHA-PQP whereas an increase in QTcF was detected 4 h after the last administration (mean difference: 29 ms [95% CI: 22, 38]; p < 0.001). These results are consistent with those obtained in the present study for the DHA-PQP fasted group.

The high-fat/high-caloric meal only had a small effect on the pharmacokinetics of DHA but PQ C_max_ increased up to 3-fold, consistently with previous report under different study conditions^17,18^. In contrast to published results that demonstrated a small or no effect of light food intake on the pharmacokinetics of PQP^19^, our data show that PQ concentrations increased following a high-fat/low-caloric meal compared with the fasted state. This apparent discrepancy may be explained by differences in the composition of the meals provided in each study. The observed QTc prolongation by DHA-PQP and A-L was likely due to PQ and lumefantrine and its desbuthyl metabolite, respectively, whereas DHA and artemether do not appear to be implicated.

Piperaquine^4^ and lumefantrine^4,20^ are known to block the hERG channel which mediates the repolarizing I_Kr_ current which is critical for cardiac repolarization. The observed increase in QTc interval after administration of these drugs was consistent with this blocking effect. However, the present study was not designed to address the clinical relevance of this QTc prolongation. It is well recognized that hERG blockade is a strong predictor of QT prolongation but not of *Torsade de Pointes*^21^. Indeed, retrospective analyses of the risk of *Torsade de Pointes* associated with drugs known to exert hERG blockade demonstrated that there is no direct correlation between the QT prolonging effect exerted by hERG blockade and *Torsade de Pointes*^22,23^.

Preclinical studies were performed to investigate the potential for DHA-PQP to elicit *Torsade de Pointes*^4^. Rabbit ventricular wedge preparations are considered a sensitive and specific experimental model for human pathological conditions characterized by a substantial reduction in repolarization reserve, a well-recognized risk factor for the potential of QT prolonging agents to trigger *Torsade de Pointes*. Different antimalarial drugs known to cause QT prolongation were compared using this model. Neither DHA-PQP nor A-L showed any effect on *Torsade de Pointes* risk score; chloroquine showed a mild risk. Dofetilide, a class III antiarrhythmic drug used as positive control, showed, as expected, a potent torsadogenic potential. This experiment also included an evaluation of early afterdepolarization. It is known that *Torsade de Pointes* is initiated by early afterdepolarization-dependent R-on-T extrasystoles; therefore, the absence of an induction of early afterdepolarization significantly reduces the probability of false positive results in terms of no arrhythmogenic effects of the tested drugs. DHA-PQP and A-L did not induce any early afterdepolarization.

In conclusion, both DHA-PQP and A-L produced significant prolongation of the QTc interval, which is a risk factor for *Torsades de Pointes*. However, current preclinical^4^ and clinical^8,24–27^ data do not suggest an increased risk of cardiac toxicity. The risk of drug-induced proarrhythmia due to QTc prolongation should further be addressed by epidemiological studies or meta-analyses. The magnitude of prolongation of the QTc interval observed with DHA-PQP was dependent on how it was administered; the smallest QTc prolongation occurred when the drug was administered in the fasted state whereas the largest QTc prolongation occurred when DHA-PQP was administered following a high-fat/high-caloric meal. Because the pharmacokinetic data obtained in the DHA-PQP fasted group were similar to those determined in a study of patients taking DHA-PQP 3 h after food intake^28^, the QTcF interval prolongation that can be experienced by patients in conditions of standard clinical care will be similar to that observed in the subjects from the DHA-PQP fasted group. Although the risk for *Torsades de Pointes* for DHA-PQP appears to be low, caution is still advised and DHA-PQP should be given to both adults and children on an empty stomach pending future research. A post-marketing observational study conducted in 10,000 patients with P. falciparum malaria of whom 10% (1,000) had a QTc monitoring did not find a clinical cardiac safety signal^8^. This was also the case in a recent large-scale phase III trial^25^.

## Methods

### Study Population

Healthy men and women aged 18–50 years with a body mass index 18–27 kg/m^2^ were included between February and August 2010. Key exclusion criteria included a history of risk factors for *Torsade de Pointes* (e.g., heart failure, hypokalemia, family history of Long QT Syndrome), the concomitant use of any other medication (except paracetamol) and any condition that might have interfered with study results. Subjects were allowed to smoke up to 5 cigarettes or equivalent per day but had to refrain from smoking while confined in the clinical center. The regular drinking of alcohol of up to 21 units (1 unit = 4 cL spirits or equivalent) for males or 14 units for females per week was allowed.

### Study Design

This was a randomized, parallel group, active- and placebo-controlled study stratified to ensure that ≥37.5% of subjects in each group were female (Clinicaltrials.gov: NCT01103830, posted 15 April 2010). Group 1 (n = 64) received placebo on Day –1 and DHA-PQP once daily for 3 days after a high-fat/low-caloric breakfast; Group 2 (n = 64) received placebo on Day –2 and A-L twice daily for 3 days from Day –1 (afternoon) to Day 3 (morning) after a high-fat/low-caloric breakfast and dinner; Group 3 (n = 40) received placebo from Day –1 to Day 3, and a single dose of 400 mg moxifloxacin on Day 4 after a high-fat/low-caloric breakfast; Group 4 (n = 40) received placebo on Day –1 and DHA-PQP once daily for 3 days after a high-fat/high-caloric breakfast. An overview of the different treatments received in each group is presented in Table 3.

**Table 3:** Treatments administered in groups 1 to 6 and the food state in each group.

Group	Day–2	Day–1	Day 1	Day 2	Day 3	Day 4	Food state
1		Placebo	DHA-PQP	DHA-PQP	DHA-PQP		High-fat/low Kcal
2	Placebo	A-L (AM)	A-L (AM and PM)	A-L (AM and PM)	A-L (AM)		High-fat/low Kcal
3		Placebo	Placebo	Placebo	Placebo	Moxifloxacin	High-fat/low Kcal
4		Placebo	DHA-PQP	DHA-PQP	DHA-PQP		High-fat/high Kcal
5		Placebo	DHA-PQP	DHA-PQP	DHA-PQP		Fasted
6		Placebo	Placebo	Placebo	Placebo	Moxifloxacin	High-fat/low Kcal

DHA = dihydroartemisinin; PQP = piperaquine phosphate; A = artemether; L = lumefantrine; AM = morning; PM = evening.

The study was performed under double blind conditions for groups 1, 3 (up to Day 4) as well as 5 and 6 (up to Day 4) morning, and in open condition for group 2 and 4. All ECG manual readings performed by Cardiabase were under blinded conditions. It is usual not to blind moxifloxacin administration in order to avoid encapsulating the tablets. Also, it was not possible to blind Groups 2 and 5 due to different dosing scheme or meal composition.

Pharmacokinetic data collected in the high fat/high caloric DHA-PQP group suggested that exposure to piperaquine as free base (PQ) was three-fold higher than previously observed in patients treated with DHA-PQP^29^. It was postulated that food intake might have increased the absorption of PQP. Therefore, two additional groups of subjects were enrolled: Group 5 (n = 40) received placebo on Day –1 and DHA-PQP once daily for 3 days in the fasted state; Group 6 (n = 20) received placebo on Day –1 to Day 3 in the fasted state, then 400 mg of moxifloxacin on Day 4 after intake of a high-fat/low-caloric breakfast. The study was double blinded for Groups 1, 3, 5 and 6 up to the morning of Day 4 and single blinded (i.e. subjects did not know whether they had received placebo or active drug) for Group 4. Group 2 received the study medication without any blinding.

Study drugs were administered as tablets containing DHA-PQP 40 mg/320 mg (Eurartesim^®^, Sigma-Tau s.p.a Industrie Farmaceutiche Riunite, Rome, Italy) and A-L 20 mg/120 mg (Riamet^®^, Novartis, Basel, Switzerland). The dose of DHA-PQP and A-L was selected based on subject’s body weight according to current recommendations.

Additional information on meals composition, blood sampling procesures, drug assays, and recommended drug dosages are shown in the supplementary file.

### ECG Assessments

Twelve-lead ECGs were recorded using a MAC5500 GE Cardiograph^®^ (GE Healthcare, Freiburg, Germany). Printouts of ECGs were taken regularly throughout the study and analysed locally to safeguard the subjects’ safety. Holter monitoring was performed on Days –1, 1, 3, and 4. Time-matched triplicate ECGs with at least 1-minute intervals were extracted at the following time-points using the expected dosing time on Day –1 and the actual dosing times on Days 1, 3 and 4: pre-dose, hourly up to 13 h and 24 h post-dose on Day –2 (Group 2), Day –1 (all other groups) and Day 3 (all groups). In addition, triplicate ECGs were recorded at 1-h intervals up to 6 h post-dose on Day 1 (Groups 1, 4 and 5) and 1, 2, 3, 4, 6, 8, 12, and 24 h post-dose on Days 1 and 4 (Groups 3 and 6). All ECG evaluations were performed without knowledge of treatment assignment.

Digitally recorded ECGs were transmitted electronically to a core ECG laboratory for computer-based, manually verified, digital caliper measurement of HR and RR, PR, QRS complex and QT intervals using the tangent method^30,31^. The core ECG laboratory personnel were blinded to treatment and all ECGs from each subject were read by the same person. Whenever possible, measurements were performed on three consecutive ECGs from a single lead, preferably lead II, and the same lead was used throughout the study for the same subject. For each subject, triplicate values at each time-point were averaged. Three different correction methods were used: Bazett, Fridericia^32^ and a population method based on a correction factor obtained by assessing the relationship between QT and RR intervals using a power model QT = αRR^β^ where α is QTc and α and β are regression parameters^33^.

### Pharmacokinetic Assessments

On Day 3 (all groups) and Day 4 (Groups 3 and 6), blood samples for the measurement of study drug concentrations were taken pre-dose, hourly up to 13 h, and 24 h post-dose. Only samples taken on Day 4 were analyzed for moxifloxacin in Group 3 and Group 6. Blood samples from Day 3 were taken to maintain blinded conditions relative to Groups 1, 4, and 5. Plasma concentrations were determined by validated reverse-phase liquid chromatography with tandem mass spectrometric detection methods. Pharmacokinetic parameters were derived from the time-concentration data by standard non-compartmental analysis using WinNonlin Professional Version 5.2 (Pharsight Corporation, Mountain View, CA, USA).

### Sample Size and Statistical Evaluations

The primary objective of this study was to demonstrate non-superiority of DHA-PQP (high-fat/low-caloric breakfast – Group 1) versus A-L (Group 2). Non-superiority was defined as an upper limit of 10 ms for the two-sided 95% confidence interval of QTcF.

Sample size calculations were based on a power of 80% and a two-sided type I error risk of 5%. For Groups 1 and 2, 64 subjects per group were considered sufficient to demonstrate non-superiority assuming a standard deviation (SD) for maximum time-matched changes from baseline in QTc of 8 ms and an expected difference of 6 ms. For Group 3, 40 subjects were considered sufficient to demonstrate a moxifloxacin effect on QTc prolongation ≥5 ms assuming an SD of 8–9 ms for time-matched changes from baseline and an expected difference of 10 ms. Forty subjects in Group 5 were considered sufficient to detect a difference of 10 ms compared with Group 1, assuming an SD of 15 ms (α = 0.05, β = 0.10). No formal statistical analysis was performed to determine the sample sizes in Groups 5 and 6 because of the large QTc prolongation seen in groups 1 and 4.

The moxifloxacin *vs*. placebo analysis was performed by comparing the mean effect in Group 3 of time-matched changes on Day 4 minus Day 1 with the mean effect in the same group of time-matched changes on Day 3 minus Day –1 using an analysis of variance (ANOVA) with terms for treatment, time and gender, including a random effect for subject. This comparison was performed within 2–8 h post-dose. Assay sensitivity was demonstrated if the lower bound of the two-sided 90% confidence interval for the difference between moxifloxacin and placebo was >5 ms; the estimated mean difference was 10 ms and the pattern of the time-effect curve of moxifloxacin was as expected.

The DHA-PQP high-fat/low-caloric meal *vs*. A-L (non-superiority) analysis was performed by comparing the mean maximum effect in Group 1 of time-matched changes on Day 3 minus Day –1 with the mean maximum effect in Group 2 of time-matched changes on Day 3 minus Day –2, using ANOVA with a term for treatment. The objective of this analysis was to evaluate whether the upper bound of the two-sided 95% confidence interval for the difference between treatments was <10 ms.

A comparison of QTcF maximum time-matched changes from baseline for DHA-PQP after a high-fat/low-caloric meal (Group 1) and under fasting conditions (Group 5) was conducted to demonstrate inferiority of DHA-PQP under fasting conditions and was performed using ANOVA with a term for treatment.

All other comparisons among the six groups were performed for QTcF in terms of 95% CI and statistical testing as described above. The effects of gender and body weight were investigated by including these terms and their interaction with treatment in the ANOVA model.

Linear regression was used to assess the relation between Cmax of PQ and the change in QTcF from baseline using data from all DHA-PQP groups (group 1, 4, and 5).

### Ethical standards

The independent Ethics Committee of Ile de France VIII, Boulogne-Billancourt, France, and the French Health Products Safety Agency, Saint-Denis, France approved the study protocol. The study was conducted in accordance with Good Clinical Practice and the principles of the Declaration of Helsinki. All subjects provided written informed consent prior to their inclusion into the study.

## Supplementary information

Consort checklist

Protocol

Supplementary Dataset 1
