# Supplementary material for: Effects of Dihydroartemisinin-Piperaquine Phosphate and Artemether-Lumefantrine on QTc Interval Prolongation
Source: Sci Rep. 2019 Jan 28;9:777. doi: 10.1038/s41598-018-37112-6 (PMC6349839; doi:10.1038/s41598-018-37112-6)
Supplement: Supplementary file 2 — Protocol [file 41598_2018_37112_MOESM2_ESM.doc]

# Synopsis

| Name of Sponsor/Company:  Sigma-Tau Industrie Farmaceutiche Riunite S.p.A. | Name of Active Ingredient:  Dihydroartemisinin / Piperaquine phosphate |
| --- | --- |
| Study Title:  Phase I, randomized, parallel group study to evaluate the effect of multiple oral doses of Eurartesim (DHA 40 mg / PQP 320 mg) on the QT/QTc interval compared to Riamet®, placebo and moxifloxacin in healthy male and female volunteers. | |
| Study Code Numbers:  Sigma-Tau Study Code: ST3073-ST3074-DM-09-006  SGS aster reference: P091198 | |
| Investigator:  Lionel Hovsepian, MD | |
| Clinical Study Center:  SGS aster s.a.s, 3 & 5, rue Eugène Million  75015 Paris, France. | |
| CRO responsible for study monitoring, safety and PK analysis:  SGS aster s.a.s, 3 & 5, rue Eugène Millon  75015 Paris, France. | |
| Laboratory responsible for ECG readings:  CardiaBase 78, avenue du XX^e^ Corps  54000 Nancy, France. | |
| Laboratory responsible for Bioanalysis:  SGS Cephac Europe SAS  90, Avenue des Hauts de la Chaume  BP. 28  86281 Saint-Benoît CEDEX  France  Phone : +33 (0)5 49 57 04 04 (standard)  Fax : +33 (0)5 49 57 22 39 | |
| Objectives:  Primary Objective:  The primary objective of the study is to evaluate the impact of a therapeutic dose of Eurartesim™ (administered with a high-fat/low-Kcal meal) compared to Riamet® (administered with a high-fat/low-Kcal meal), after multiple dose administration for 3 days in healthy male and female subjects on QTcF.  A single oral dose of 400 mg of moxifloxacin will be used as a positive control in order to document the sensitivity of the experimental conditions.  Secondary Objectives:  The secondary objectives are:   - To evaluate the impact of a therapeutic dose of Eurartesim™ under different food intake conditions on the cardiac activity, as expressed by ECG parameters. - To assess the safety and tolerability of Eurartesim™ under different food intake conditions in healthy subjects. - To evaluate the absolute QT prolongation of Eurartesim™ at therapeutic dose under different food intake conditions. - To evaluate the relationship between the PK parameters of DHA and PQ and the ECG parameters. - To evaluate the impact of high-fat/high-Kcal or high-fat/low-Kcal meals on the overall relative bioavailability of Eurartesim™. - To compare, within the Eurartesim™ groups, the PK profiles and the QT intervals of subjects with a body weight below/above 75kg. | |
| Methodology/Study Design:  This will be a single center, randomized, placebo-, active- and comparator-controlled, parallel, 4 groups study in 208 healthy subjects stratified by gender. This study will be double blind for the Eurartesim™ (high-fat/low-Kcal meal) *vs.* placebo and open for the other groups.  ECG readings will be performed in blind conditions. | |
| Number of Subjects:  Planned: 208 split as follows: 64 in group 1 and group 2, 40 in group 3 and group 4 (see below).  An interim analysis will be carried-out after completion of 50% of the subjects (*i.e.* 32 subjects in group 1 and in group 2) in order to control that the assumption on the variability used for the initial sample size determination was correct. | |
| Diagnosis and Main Criteria for Inclusion:  The study will be carried out in healthy male and female Caucasian volunteers, aged 18 to 50 (inclusive) years old. | |
| Duration of Treatment:  3 days (plus one day of placebo) for group 1 and group 4.  3.5 days (plus one day of placebo) in group 2.  4 days of placebo and one day of moxifloxacin in group 3 | |
| Dosage regimen:  Group 1 (64 subjects)   - 3 or 4 tablets of Eurartesim^TM^ placebo, depending of body weight, on Day -1 morning - 3 or 4 tablets of Eurartesim™, depending of body weight, once daily from Day 1 to Day 3   Administration of placebo and Eurartesim will be in fed condition following a high-fat/low-Kcal meal (*ca.*  400 Kcal)  From Day -1 to Day 3, each administration will be separated by an interval of 24 ± 0.5 h.  Group 2 (64 subjects)   - 3 or 4 tablets of Eurartesim™ placebo on Day -2 morning - 4 tablets of Riamet® on Day -1 evening, 8 h before the first administration of Day 1 - 4 tablets of Riamet® bid (with an interval of 12 ± 0.5 h) on Day 1 and Day 2 - 4 tablets of Riamet® in the morning of Day 3.   All the administrations of placebo and Riamet® will be in fed condition, following a high-fat/low-Kcal meal (*ca.*  400 Kcal).  Group 3 (40 subjects)   - 3 or 4 tablets of Eurartesim™ placebo. Administration will be in fed condition following a high-fat/low-Kcal meal (*ca.*  400 Kcal) once daily from Day -1 to Day 3 - 1 tablet of Izilox® (400 mg moxifloxacin) in fasting condition on Day 4 morning.   From Day -1 to Day 4, each administration will be separated by an interval of 24 ± 0.5 h.  Group 4 (40 subjects)   - 3 or 4 tablets of Eurartesim™ placebo, depending of body weight, on Day -1 morning - 3 or 4 tablets of Eurartesim™, depending of body weight, once daily from Day 1 to Day 3   All the administrations of placebo and Eurartesim™ will be in fed condition, following a high-fat/high-Kcal meal (*ca.*  1000 Kcal).  From Day -1 to Day 3, each administration will be separated by an interval of 24 ± 0.5 h.  For groups 1, 3 and 4, the number of tablets of Eurartesim™ or Eurartesim™ placebo to be given will be adapted according to the body weight as follows:   - Body weight < 75 kg 3 tablets - Body weight ≥ 75 kg 4 tablets   In this protocol the definitions “Eurartesim™ placebo” and “placebo” will be considered synonymous. | |
| Criteria for Evaluation: | |
| Efficacy:  Not applicable. | |
| Pharmaco- and cardiogenetics:  Two blood samples of 2x 6 mL will be drawn on Day –1 for groups 1, 3 and 4 and on Day -2 on group 2.  One will be used for the determination of genetic polymorphisms of CYP450 2D6, 3A4, 3A5, 3A7 and potentially of other metabolic enzymes/transporters (*e.g.* other CYP450, P-gp, BSEP, MRP2, OATPs, UGT1A1, NTCP) in subjects receiving Eurartesim™.  One will be used for the determination of the genetic polymorphism of KVLQT1, HERG, SCN5A, and KCNE1. This assessment may be performed only for subjects who will be found to exhibit prolonged QT interval during the course of the study or abnormal variation of QT across time.  PK:  Group 1 and Group 4  Blood samples for assessment of DHA and PQ plasma levels will be taken at the following time points:   - Day 1 : pre-dose, 1, 2, 3, 4, 5, 6 h post-dose - Day 3 : pre-dose, 1, 2, 3, 4, 5, 6, 7, 8, 9, 10, 11, 12, and 13 h post-dose - Day 4 : 24 and 36 h after the last administration (Day 3 morning) (only PQ) - Day 5 : 48 and 60 h after the last administration (Day 3 morning) (only PQ) - Day 6 : 72 h after the last administration (Day 3 morning) (only PQ) - Day 7 : 96 h after the last administration (Day 3 morning) (only PQ) - Day 8 : 120 h after the last administration (Day 3 morning) (only PQ) - Day 10 : 168 h post-dose. (only PQ)   A total of 29 blood samples, and an approximate volume of 183 mL, will be collected from each subject. | |
|  | |
| Group 2  Blood samples for assessment of artemether, DHA and lumefantrine plasma levels will be taken at the following time points:   - Day -1 : pre-dose - Day 3 : pre-dose, 1, 2, 3, 4, 5, 6, 7, 8, 9, 10, 11, 12, and 13 h post-dose - Day 4 : 24 and 36 h after the last administration (Day 3 morning) (only lumefantrine) - Day 5 : 48 and 60 h after the last administration (Day 3 morning) (only lumefantrine) - Day 6 : 72 h after the last administration (Day 3 morning) (only lumefantrine) - Day 7 : 96 h after the last administration (Day 3 morning) (only lumefantrine) - Day 8 : 120 h after the last administration (Day 3 morning) (only lumefantrine) - Day 10 : 168 h post-dose (only lumefantrine).   A total of 23 blood samples, and an approximate volume of 141 mL, will be collected from each subject.  Group 3  Blood samples for assessment of moxifloxacin plasma levels will be taken at the following time points:   - Day 1 : pre-dose, 1, 2, 3, 4, 5, 6 h post-dose - Day 3 : pre-dose, 1, 2, 3, 4, 5, 6, 7, 8, 9, 10, 11, 12, and 13 h post-dose - Day 4 : pre-dose (corresponding to 24 h post-dose (Day 3 dosing)), 1, 2, 3, 4, 6, 8, 12, and 24 h post-dose.   A total of 30 blood samples, and an approximate volume of 188 mL, will be collected from each subject. | |
| QT:  QT intervals will be manually calculated. Triplicate ECG with at least a 1 min interval will be extracted from the core ECG laboratory at the following time points:  **Group 1 and Group 4**   - Day -1 : pre-dose, 1, 1.5, 2, 2.5, 3, 4, 5, 6, 7, 8, 9, 10, 11, 12, and 13 h post-dose - Day 1 : pre-dose (corresponding to a 24 h post-dose of Day-1), 1, 2, 3, 4, 5, and 6 h post-dose (holter will remain till 24 h to maintain double blind versus group 3) - Day 3 : pre-dose, 1, 1.5, 2, 2.5, 3, 4, 5, 6, 7, 8, 9, 10, 11, 12, 13, and 24 h post-dose.   **Group 2**   - Day -2 : pre-dose, 1, 2, 3, 4, 5, 6, 7, 8, 9, 10, 11, 12, 13, and 24 h post-dose - Day 3 : pre-dose, 1, 2, 3, 4, 5, 6, 7, 8, 9, 10, 11, 12, 13, and 24 h post-dose.   **Group 3**   - Day -1 : pre-dose, 1, 1.5, 2, 2.5, 3, 4, 5, 6, 7, 8, 9, 10, 11, 12, and 13 h post-dose - Day 1 : pre-dose (corresponding to a 24 h post-dose), 1, 2, 3, 4, 6, 8, 12 and 24 h post-dose - Day 3 : pre-dose, 1, 1.5, 2, 2.5, 3, 4, 5, 6, 7, 8, 9, 10, 11, 12, and 13 h post-dose - Day 4 : pre-dose (corresponding to 24 h post-dose of Day 3), 1, 2, 3, 4, 6, 8, 12, and 24 h post-dose. | |
| Safety:  Monitoring for the occurrence of adverse events.  Changes in physical examination, vital signs (blood pressure and pulse rate), ECG and clinical laboratory tests (biochemistry, hematology, and urinalysis). | |
| Statistical Methods:  ECG analysis:  This analysis will be performed for all the ECG variables (QT, QTcB, QTcF, QTcP, RR, PR and QRs) in the **QT/QTc prolongation analysis set** (*i.e.* all subjects who received the study treatments as planned in the protocol, and having reliable holter-extracted triplicate ECG recordings on baseline and on Day 3 or 4 depending on group).  The ECG data (triplicate) will be summarized in terms of means by subject, day and time-point.  Two different endpoints will be computed as follows:   - maximum of the time-matched changes from baseline (primary) - mean of the time-matched changes from baseline (secondary).   Primary analyses (to be performed in the listed order) will be two, both based on QTcF:  Analysis described at point 1 is a sensitivity analysis to validate the study methodology and conduction.  **1. Comparison Izilox**® ***vs.* placebo,** in order to demonstrate superiority of Izilox® *vs.* placebo (assay sensitivity) by at least 5 ms and with a mean effect above 10 ms. This will be obtained by comparing the mean effect in group 3 of (time-matched changes Day 4 – Day 1) with the mean effect in group 3 of (time-matched changes Day 3 – Day -1) by means of an ANOVA model with terms for treatment, time and gender, including a random effect for subject.  **2. Comparison Eurartesim^TM^ (high-fat/low-Kcal meal) *vs.* Riamet®,** in order to demonstrate non-superiority of Eurartesim^TM^ (high-fat/low-Kcal meal) *vs.* Riamet® with an upper limit of 10 ms and an expected difference in means of 6 ms. This will be obtained by comparing the mean effect in group 1 of time-matched changes Day 3 – Day -1, summarized by subject through the maximum value, with the mean effect in group 2 of time-matched changes Day 3 – Day -2, summarized by subject through the maximum value, by means of an ANOVA model with terms for treatment and gender.  Assay sensitivity (analysis 1) will be demonstrated if the lower bound of the two-sided 90% confidence interval for the difference between treatments is >5 ms; if the estimated mean difference is >10 ms and if the pattern of the time-effect curve of Izilox® is as expected.  The objective of analysis 2 will be demonstrated if the upper bound of the two-sided 95% confidence interval for the difference between treatments is <10 ms.  Secondary analyses:  **3. All possible comparisons between Riamet® (group 2), placebo (group 3) and Eurartesim^TM^ after high-fat/high-Kcal meal (group 4)** will be performed for QTcF on the primary endpoint, as specified for the primary analysis 2.  **4. All possible comparisons between Riamet® (group 2), placebo (group 3) and Eurartesim^TM^ (groups 1 and 4)** will be performed for QT, QTcB, QTcP, RR, PR and QRs on the primary endpoint, as specified for the primary analysis 2.  **5. All possible comparisons between Riamet® (group 2), placebo (group 3) and Eurartesim^TM^ (groups 1 and 4)** will be performed for all the ECG variables on the secondary endpoint, as specified for the primary analysis 2.  **6. Comparisons between Izilox**® ***vs.* placebo (group 3)**, to be performed for QT, QTcB, QTcP, RR, PR and QRs on the time-matched changes from baseline, as specified for the primary analysis 1.  **7. Estimation of the pattern of the effect of each treatment across time**, by means of an ANOVA model for repeated measures, assessed on the time-matched changes from baseline over the collection period for all the ECG variables and including data collected for Eurartesim® given in both conditions (*i.e.* high-fat/low-Kcal meal and high-fat/high-Kcal meal), Riamet®, and placebo.  **8. Categorical analysis for all the QTc variables**, providing incidence and percentage of subjects, by treatment, presenting at least one QTc value above pre-specified threshold.  **9. Morphological analysis for all the QTc variables**, providing incidence and percentage of subjects, by treatment, presenting at least one abnormality.  **10. Scatter-plots of the time-matched plasma concentrations *vs.* QTcF values** (DHA and PQ separately, group 1 and 4 and artemether, DHA and lumefantrine).  Safety analysis:  Summary descriptive statistics will be provided for observed values and changes from baseline values by group, day, and by gender and overall, for all the safety and laboratory parameters in the **Safety analysis set** (*i.e.*all subjects who received at least one dose of any study treatment). This analysis will include vital signs and ECG parameters, body weight, BMI, physical examination and hematology/biochemistry parameters. AEs will be summarized by group, MedDRA system organ class and preferred term.  PK analysis:  Summary descriptive statistics (n, arithmetic mean, SD, CV% and geometric mean) will be provided by group, compound and day (overall and by gender) for plasma concentrations, and by group and compound (overall and by gender) for PK parameters. All the analyses will be performed in the **PK analysis set** (*i.e.* all subjects for whom blood sample collection was performed without major protocol deviation). PK parameters will include C_max_, t_max_, AUC_0-t_, AUC_0-∞_ and t_1/2_.  Graphic displays of plasma concentrations will also be provided.  The possible impact of food and body weight (below and equal or above 75 kg) effects will be assessed. | |
