# Supplementary material for: Effects of Dihydroartemisinin-Piperaquine Phosphate and Artemether-Lumefantrine on QTc Interval Prolongation
Source: Sci Rep. 2019 Jan 28;9:777. doi: 10.1038/s41598-018-37112-6 (PMC6349839; doi:10.1038/s41598-018-37112-6)
Supplement: Supplementary file 3 — Supplementary Dataset 1 [file 41598_2018_37112_MOESM3_ESM.docx]

**SUPPLEMENTARY DATA**

**Effects of Dihydroartemisinin-Piperaquine Phosphate and Artemether-Lumefantrine on QTc Interval Prolongation**

Christian Funck-Brentano^1^ ⚫ Antonella Bacchieri^2^ ⚫ Giovanni Valentini^2^ ⚫ Silvia Pace^2^ ⚫ Silva Tommasini^2^ ⚫ Pascal Voiriot^3^ ⚫ David Ubben^4^ ⚫ Stephan Duparc^4^ ⚫ Eric Evene^5^ ⚫ Mathieu Felices^6^ ⚫ Marco Corsi^2^

^1^ INSERM, CIC-1421 and UMR ICAN 1166, Sorbonne Université, Faculty of Medicine, AP-HP, Pitié-Salpêtrière Hospital, Department of Pharmacology and Clinical Investigation Center, Institute of Cardiometabolism and Nutrition (ICAN), F-75013 Paris, France.

^2^ Sigma-tau Industrie Farmaceutiche Riunite S.p.A., Pomezia (Rome), Italy

^3^ Cardiabase, Nancy, France

^4^ Medicines for Malaria Venture, Geneva, Switzerland

^5^ SGS-Aster, Paris, France

^6^ PhinC development, Evry, France

[Blood sampling procedures for piperaquine, artemether, dihydroartemisinin, lumefantrine, desbutyl-lumefantrine and moxifloxacin 2](#_Toc526457743)

[Bioanalytical methods 2](#_Toc526457744)

[Meals composition and timing relative to drug administrations 5](#_Toc526457745)

[Placebo composition 6](#_Toc526457746)

[Current EMA recommendations and WHO recommendations before 2015 based on body weight for DHA-PQP at the time of the present study. 6](#_Toc526457747)

[Plasma concentrations vs. time profile of piperaquine, lumefantrine and desbutyl-lumefantrine 7](#_Toc526457748)

[Relationship between piperaquine (PQ) concentration (Cmax and AUC) and change in QTcF 8](#_Toc526457749)

#

# Blood sampling procedures for piperaquine, artemether, dihydroartemisinin, lumefantrine, desbutyl-lumefantrine and moxifloxacin

Blood was collected into Vacutainer (4.5 mL) tubes containing lithium heparin anticoagulant; tubes were gently inverted 180° at least 10 times and immediately placed on ice; within 30 min from collection (1 h for moxifloxacin), blood samples were centrifuged at 1000 x g, 15 minutes at 4°C (1900 x g, 10 minutes at 4°C for moxifloxacin). Plasma aliquots were transferred to polypropylene cryovial tubes and frozen at -75±10°C until shipment to bioanalytical laboratory.

Plasma study samples were transferred to bioanalytical facilities packaged with sufficient dry ice to ensure that samples remained deep frozen during the shipment. Upon arrival at bioanalytical laboratory, the plasma samples were immediately stored in a freezer at -75±10°C until analysis within 6 months.

# Bioanalytical methods

Plasma samples were analyzed for piperaquine free base, artemether, dihydroartemisinin, lumefantrine, desbutyl-lumefantrine and moxifloxacin concentrations by SGS Cephac Europe (Saint Benoit, France).

All analyte concentrations were quantified by high-performance liquid chromatography-tandem mass spectrometry (HPLC-MS/MS) methods validated according to US Food and Drug Administration (FDA) guidelines. Validation studies and study sample analyses were conducted according to the principles of Good Laboratory Practice (GLP).

Piperaquine and the internal standard (deuterated piperaquine) were purified from plasma by a robotized solid phase extraction on Oasis HLB µElution plate followed by chromatographic separation and MS/MS detection. Aliquots of 50 µL of human plasma were used. A Phenomenex Kinetex 2.6 µm C18 100 Å, 50 x 2.1 mm column at about 55°C was used for chromatographic separation under gradient conditions at a 0.7 mL/min flow rate. Mobile phase (MP) A was 20 mM of ammonium formate containing 0.5% of formic acid7acetonitrile (49/1 v/v), and MP B was 0.5 % formic acid in acetonitrile. Piperaquine and its internal standard were monitored by an MS/MS detector in positive multiple reaction monitoring (MRM) mode. The single charged Q1/Q3 transitions were 535.4/288.2 atomic mass units (amu) for piperaquine and 541.3/294.2 amu for the internal standard. The typical retention time was 2.7 min for both analytes. The lower limit of quantification (LLOQ) was 5.0 ng/mL, and the calibration curve range was 5.0-500 ng/mL (8 calibration standards in duplicate, weighting factor 1/x^2^). Independent quality-control samples had concentrations of 15.0, 200.0 and 400 ng/mL of piperaquine. Samples were analyzed for piperaquine determination in a total of 49 analytical runs (46 accepted). The linearity and reproducibility of the calibration curves were evaluated from repeated analysis of the calibration curve samples. The inter-assay accuracy and precision were within ± 3 % and < 4 %, respectively. The mean R^2^ value was > 0.98. The accuracy and precision evaluated from repeated analysis of the quality-control samples were within ± 3 % and < 5 %, respectively.

Artemether, dihydroartemisinin and their respective internal standards (deuterated artemether and artemisinin respectively) were purified from plasma by liquid/liquid extraction followed by chromatographic separation and MS/MS detection. All sample processing was carried out in ice, i.e. from thawing through addition of the extraction solvent. Aliquots of 200 µL of human plasma were used. A Waters Atlantis dC18 (2.1 x 150 mm; 5 µm) column at about 40°C was used for chromatographic separation under gradient conditions, the mobile phases being MP A: 10 mM ammonium acetate with 0.1% of formic acid/methanol/acetonitrile; 34/33/33 (v/v/v) and MP B: acetonitrile/water 95/5 (v/v) with 0.1% formic acid.

Artemether, dihydroartemisinin and their respective internal standards were monitored by an MS/MS detector in positive MRM mode. The single charged Q1/Q3 transitions were 316.1/267.3 amu for artemether; 319.0/267.2 amu for deuterated artemether; 302.2/267.2 amu for dihydroartemisinin and 300.1/209.3 amu for artemisinin. The typical retention times were 8.5 min for artemether and its internal standard; 3.8 and 4.5 min for dihydroartemisinin and its internal standard, respectively. The LLOQ was 10.0 ng/mL, and the calibration curve range was 10.0-1500 ng/mL (8 calibration standards in duplicate, weighting factor 1/x^2^), for both analytes. Independent quality-control samples had concentrations of 20.0, 450 and 1100 ng/mL of both artemether and dihydroartemisinin. Samples were analyzed for both artemether and dihydroartemisinin determinations in a total of 20 analytical runs (18 and 17 accepted for artemether and dihydroartemisinin, respectively) and for dihydroartemisinin determination only in a total of 46 analytical runs (40 accepted). The linearity and reproducibility of the calibration curves were evaluated from repeated analysis of the calibration curve samples. The mean R^2^ value was > 0.98. For artemether determination, the inter-assay accuracy and precision were within ± 2% and < 5%, respectively. The accuracy and precision evaluated from repeated analysis of the quality-control samples were within ± 5% and < 10%, respectively. For dihydroartemisinin determination, the inter-assay accuracy and precision were within ± 4% and < 8%, respectively. The accuracy and precision evaluated from repeated analysis of the quality-control samples were within ± 6% and < 8%, respectively.

Lumefantrine, desbutyl-lumefantrine and their respective internal standards (deuterated lumefantrine, and deuterated desbutyl-lumefantrine, respectively) were purified from plasma by liquid/liquid extraction at pH 3 followed by chromatographic separation and MS/MS detection. Aliquots of 100 µL of human plasma were processed. A Phenomenex Kinetex 2.6 µm C18 100 Å, 50 x 2.1 mm column at about 60°C was used for chromatographic separation under gradient conditions at a 0.8 mL/min flow rate, the mobile phases being MP A: 20 mM ammonium formate with 0.5% of formic and MP B: acetonitrile with 0.5% formic acid. Lumefantrine, desbutyl-lumefantrine and their respective internal standards were monitored by an MS/MS detector in positive MRM mode. The single charged Q1/Q3 transitions were 528.2/510.2 amu for lumefantrine; 472.1/454.2 amu for desbutyl-lumefantrine; 546.4/527.4 amu for deuterated lumefantrine and 481.4/463.2 amu for deuterated desbutyl-lumefantrine. The typical retention times were 1.7 min for lumefantrine and its internal standard; 0.8 min for desbutyl-lumefantrine and its internal standard. The LLOQ was 50.0 ng/mL and 5.00 ng/ml for lumefantrine and desbutyl-lumefantrine respectively, and the calibration curve ranges were 50.0-8000 ng/mL for lumefantrine and 5.00-100 ng/mL for desbutyl-lumefantrine (8 calibration standards in duplicate, weighting factor 1/x^2^), for both analytes. Independent quality-control samples had concentrations of 150/15.0, 3000/50.0 and 7000/75.0 ng/mL of lumefantrine/desbutyl-lumefantrine. Samples were analyzed for both lumefantrine and desbutyl-lumefantrine determinations in a total of 23 analytical runs (17 accepted). The linearity and reproducibility of the calibration curves were evaluated from repeated analysis of the calibration curve samples. The mean R^2^ value was > 0.98. For lumefantrine and desbutyl-lumefantrine determinations, the inter-assay accuracy and precision were within ± 3% and < 7%, respectively. The accuracy and precision evaluated from repeated analysis of the quality-control samples were within ± 8% and < 10%, respectively.

Moxifloxacin and the internal standard (pefloxacin) were purified from plasma by a robotized solid phase extraction on Oasis HLB cartridges followed by chromatographic separation and MS/MS detection. Aliquots of 250 µL of human plasma were processed.

A Thermo BSD Hypersil 3 µm 100 x 4 mm column at about 40°C was used for chromatographic separation under isocratic conditions at a 1.0 mL/min flow rate. Moxifloxacin and the internal standard were monitored by an MS/MS detector in positive MRM mode. The single charged Q1/Q3 transitions were 402.2/384.2 amu for moxifloxacin and 334.2/290.2 amu for the internal standard. The typical retention times were 2.5 and 1.4 min for moxifloxacin and the internal standard, respectively. The LLOQ was 25.0 ng/mL, and the calibration curve range was 25.0-5000 ng/mL (8 calibration standards in duplicate, weighting factor 1/x^2^). Independent quality-control samples had concentrations of 75.0, 1500.0 and 4000 ng/mL of moxifloxacin. Samples were analysed for moxifloxacin determination in a total of 14 analytical runs (12 accepted). The linearity and reproducibility of the calibration curves were evaluated from repeated analysis of the calibration curve samples. The mean R^2^ value was > 0.98. The inter-assay accuracy and precision were within ± 3 % and < 6 %, respectively. The accuracy and precision evaluated from repeated analysis of the quality-control samples were within ± 6 % and < 7 %, respectively.

Stability of artemether and DHA in human plasma at ‑75°C±10°C has been demonstrated for up to 190 days. This stability period covers the length of time from specimen collection (first sample collected on 23-Feb-2010) to analysis (last sample analysed on 27-Aug‑2010) i.e., 185 days.

Stability of piperaquine in human plasma at ‑75°C±10°C has been demonstrated for up to 211 days. This stability period covers the length of time from specimen collection (first sample collected on
23-Feb-2010) to analysis (last sample analysed on 18-Aug‑2010) i.e., 176 days.

Stability of lumefantrine and desbutyl-lumefantrine in human plasma at ‑75°C±10°C has been demonstrated for up to 119 days. This stability period covers the length of time from specimen collection (first sample collected on 23-Feb-2010) to analysis (last sample analysed on 10-Jun‑2010) i.e., 107 days.

Stability of moxifloxacin in human plasma at ‑75°C±10°C has been demonstrated for up to 203 days. This stability period covers the length of time from specimen collection (first sample collected on 23-Feb-2010) to analysis (last sample analysed on 10-Aug‑2010) i.e., 168 days.

# Meals composition and timing relative to drug administrations

| **High-fat/low-Kcal menu** |
| --- |
| 25 g of cashews |
| 250 mL of full-fat milk |
| **Total energy content:**            *ca.* 347 Kcal |
| **Total fat content:**                   *ca.* 20 g (57% of total calories) |
| **Total protein:**                          *ca.* 11 g (17% total calories) |
| **Total carbohydrate:**              *ca.* 23 g (26% of total calories) |

| **High-fat/high-Kcal meal menu** |
| --- |
| Two eggs (fried in blended oil) |
| Bacon equivalent (grilled) |
| One slice of white toast with 10 g butter (2 pats) |
| 2 hash brown potatoes |
| 240 mL full-fat milk |
| **Total energy content:**            *ca.* 963 Kcal |
| **Total fat content:**                   *ca.* 75 g (62% of total calories) |
| **Total protein:**                          *ca.* 42 g (17% total calories) |
| **Total carbohydrate:**              *ca.* 53 g (21% of total calories) |

For group 5 and group 6, the breakfast served from Day -1 to Day 3 consisted of two slices of toasted bread, 25 g. of preserve, 75 mL of milk - 5% fat, one cup of coffee and one spoon of sugar.

All breakfasts were given in the morning just before dosing. Meal times were identical between groups and comparisons with placebo were always performed between groups receiving identical meals.

# Placebo composition

Lactose monohydrate 259.8 mg, mycrocristalline cellulose 100 mg, pregelatinised starch 100 mg, dextrin 22 mg, croscarmellose sodium 23.6 mg, hypromellose 4.8 mg, magnesium stearate 4.8 mg, Opadry white 03B28796© (film coating) 15 mg. Tablets formulation.

# Current EMA recommendations and WHO recommendations before 2015 based on body weight for DHA-PQP at the time of the present study.

Dosing should be based on body weight as shown in the table below:

| Body weight (kg) | Daily dose (mg) | | Tablet strength and number of tablets per dose |
| --- | --- | --- | --- |
|  | PQP | DHA |  |
| 5 to <7 | 80 | 10 | ½ x 160 mg / 20 mg tablet |
| 7 to <13 | 160 | 20 | 1 x 160 mg / 20 mg tablet |
| 13 to <24 | 320 | 40 | 1 x 320 mg / 40 mg tablet |
| 24 to <36 | 640 | 80 | 2 x 320 mg / 40 mg tablets |
| 36 to <75 | 960 | 120 | 3 x 320 mg / 40 mg tablets |
| 75 to 100 | 1,280 | 160 | 4 x 320 mg / 40 mg tablets |
| >100 | There are no data on which to base a dose recommendation in patients weighing >100 kg. | | |

**Current WHO recommendations (since 2015) based on body weight for DHA-PQP (WHO Malaria Treatment Guidelines 3^rd^ Edition, 2015).**

| Body weight (Kg) | Dihydroartemisinin + piperaquine dose (mg) given daily for 3 days |
| --- | --- |
| 5 to < 8 | 20 + 160 |
| 8 to < 11 | 30 + 240 |
| 11 to < 17 | 40 + 320 |
| 17 to < 25 | 60 + 480 |
| 25 to < 36 | 80 + 640 |
| 36 to < 60 | 120 + 960 |
| 60 to < 80 | 160 + 1280 |
| > 80 | 200 + 1600 |

**Current WHO recommendations and recommendations at the time of the study based on body weight for A-L (WHO Malaria Treatment Guidelines 3^rd^ Edition, 2015).**

*Target dose range:* A total dose of 5–24 mg/kg bw of artemether and 29–144 mg/kg bw of lumefantrine

*Recommended dosage regimen:* Artemether + lumefantrine is given twice a day for 3 days (total, six doses). The first two doses should, ideally, be given 8 h apart:

| Body weight (kg) | Dose (mg) of artemether + lumefantrine given twice daily for 3 days |
| --- | --- |
| 5 to < 15 | 20 + 120 |
| 15 to < 25 | 40 + 240 |
| 25 to < 35 | 60 + 360 |
| >= 35 | 80 + 480 |

# Plasma concentrations vs. time profile of piperaquine, lumefantrine and desbutyl-lumefantrine


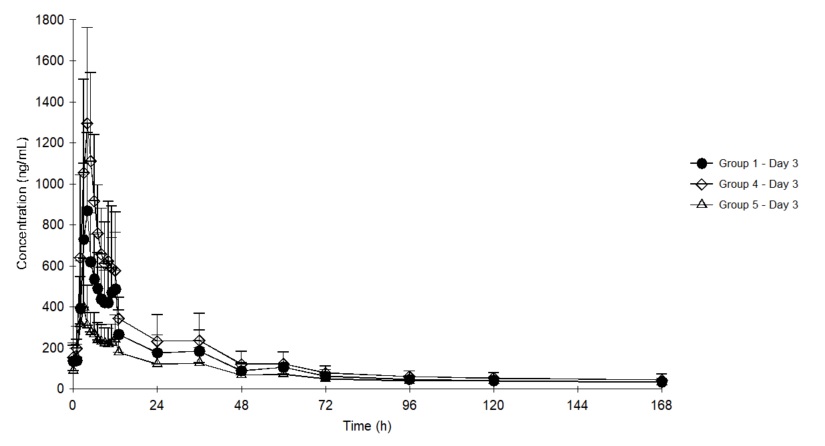


Piperaquine


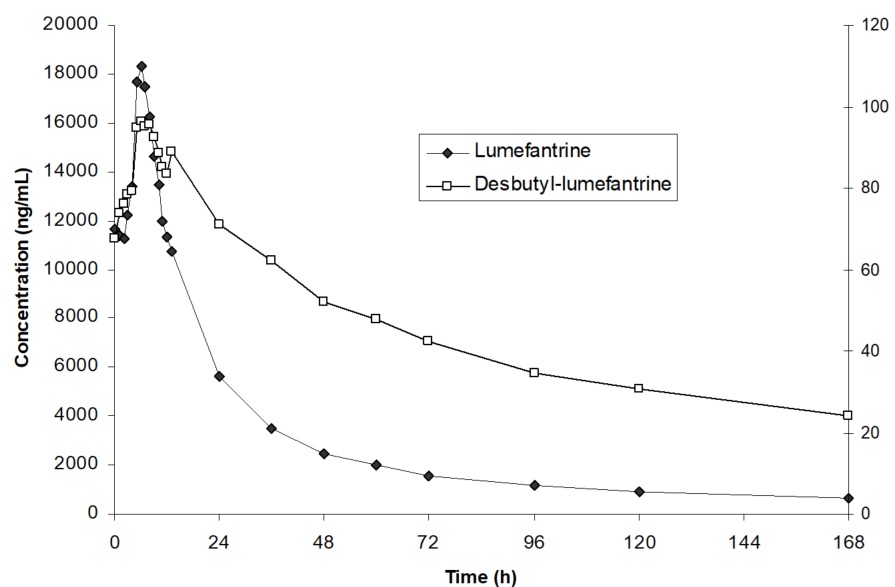


Lumefantrine

Desbutyl-lumefantrine

# Relationship between piperaquine (PQ) concentration (Cmax and AUC) and change in QTcF

***QTcF maximum change (ms) vs PQ Cmax (ng/mL)***

***QTcF average change (ms) vs PQ AUC0-24 (ng.h/mL)***
